# Biomimetic, Highly Reusable and Hydrophobic Graphene/Polyvinyl Alcohol/Cellulose Nanofiber Aerogels as Oil-Removing Absorbents

**DOI:** 10.3390/polym14061077

**Published:** 2022-03-08

**Authors:** Peiyuan Feng, Xiwen Wang, Jin Yang

**Affiliations:** School of Light Industry and Engineering, South China University of Technology, Guangzhou 510640, China; fengpeiyuan138@163.com

**Keywords:** aerogel, bidirectional freezing, biomimetic structure, oil absorption, recyclability

## Abstract

Aerogels have great potential in oil absorption applications; however, many reported aerogels have the drawbacks of a low oil-recovery rate and poor mechanical properties, which limit their application. In this study, highly reusable graphene oxide (GO)/TEMPO-oxidized cellulose nanofiber (TOCN)/polyvinyl alcohol (PVA) aerogels with excellent mechanical properties and with an architecture similar to that of *Thalia dealbata* stems were fabricated through a three-step process of bidirectional-freezing, freeze-drying, and chemical vapor deposition (CVD) modification. After CVD modification, the modified GTPA (MGTPA) accorded hydrophobicity. The synergistic effects of the three components and the unique biomimetic structure conferred biomimetic-MGTPA (b-MGTPA) with excellent compressible properties. As an adsorbent, b-MGTPA showed a high adsorption capacity (75–151 g/g) for various types of organic solvents. In addition, its high compressibility enables b-MGTPA to have fast and highly efficient recovery of absorbed oil through simple mechanical squeezing and it possesses excellent reusable stability (the oil recovery rate and oil retention rate reached 80% and 91.5%, respectively, after 10 repeated absorption–compression cycles).

## 1. Introduction

In the process of petroleum exploration, exploitation, transportation, and processing, oil-spill accidents may occur due to accidents or for technical reasons, causing serious impacts on the environment [1,2,3]. Aerogels, as ideal absorbents, can effectively and selectively absorb oil in an oil–water mixture due to their high adsorption rate, large specific surface area, and their light weight; however, many aerogels still face many problems, such as a low mechanical strength, low oil absorption capacity, and low recyclability. In particular, the oil recovery rate and reusability of absorbents restricts the extensive application of oil–water-separation materials [4,5,6].

Cellulose, as a natural low-cost material, has been attracting more attention from researchers due to its wide availability, biocompatibility, and good flexibility. It has been widely used to prepare ultralight absorbent aerogel materials [7,8,9]. However, most cellulose-based aerogels have the drawback of having a brittle mechanical performance, which causes irreversible deformation during the recycling of the oil absorption process. To overcome the drawbacks of cellulose aerogels, many functional nanomaterials have been used as reinforcing materials in combination with nanocellulose to improve the mechanical properties of aerogel, such as polyimide [10], poly(N-isopropylacrylamide) [11], and polyvinyl alcohol (PVA) [12]. Especially, PVA with long polymer chains is of benefit in the formation of high-density hydrogen bonding with nanocellulose, which can enhance the mechanical properties of the aerogel, forming strong interactions with each other [13]. Therefore, we consider using PVA as a reinforcement to enhance the mechanical properties of aerogels. Graphene oxide (GO) contains a large number of oxygen-containing functional groups on its surface, a large specific surface area, excellent mechanical properties, and has an excellent dispersion in solution, making it an ideal enhancement phase for aerogel materials. Nanocellulose, as the dispersant, can prevent the self-agglomeration of GO. Thus, we considered adding GO to the aerogel. However, the obtained aerogel has a random structure, which is more likely to rupture, resulting in inelastic deformation during compression [14,15], which shows that there is still a great deal of room for improvement.

Natural organisms often provide inspiration for the design of compressible materials [16]. The compressibility of some natural materials is attributed to their complex multi-scale architectures with their long-range orders [17,18,19]. Based on previous reports, these three-dimensional porous lamellar layer structures show better mechanical properties during compression. Recently, Yang [20] prepared highly compressible biomass-derived nanofiber aerogel with an ordered honeycomb structure via directional freeze drying. Gao [21] obtained hyper-elastic carbon–graphite aerogel with numerous arched parallel lamellar structures through bidirectional freeze drying. Based on previous work, it has been determined that the microstructure of aerogels is essential to their performance.

Inspired by natural *Thalia dealbata*, which has a unique architecture with lamellar layers interconnected by bridges [22]. a combination of bidirectional freezing and the addition of reinforcing materials was employed in the design of a highly compressive graphene/polyvinyl alcohol/cellulose nanofiber aerogel. Then, CVD modification was carried out using simple sialylation to gain hydrophobicity. After modification, the aerogel has an excellent oil–water separation effect for an oil–water mixture and its maximum absorption capacity ranges from 75 to 151 times its own weight (for different organic solvents). Moreover, b-MGTPA exhibits excellent reusability, maintaining a good absorption capacity, even after 10 distillation or extrusion cycles. The b-MGTPA retrieves more than 90% of oil by mechanical squeezing (oil recovery rate and oil retention rate reached 80% and 91.5%, respectively, after 10 absorption–compression cycles) due to its unique biomimetic structure. We believe that b-MGTPA has great potential application in practical oil or organic solvent leakage treatments.

## 2. Experimental Section

### 2.1. Materials

Graphene oxide dispersion was purchased from Suzhou Tanfeng Graphene Co., Ltd. (Suzhou, China). Conifer wood pulp fiber was received from Arauco Paper Co., Ltd. (Santiago, Chile). Polydimethylsiloxane (PDMS, Sylgard 184, Dow corning, Midland, MI, USA), Analytical grade PVA (Mw: 95,000 g/mol, alcoholysis degree: 99%) and all other chemical reagents were purchased from Aladdin Chemical Reagent, Co., Ltd. (Shanghai, China). All water used in the experiments was deionized water and all reagents did not require further treatment.

### 2.2. Preparation of TOCN Suspension

TEMPO-oxidized CNFs (TOCN) were prepared using a previously reported method [23]. Conifer wood pulp fibers (1.0 g) were put into a blender and 50 mL of deionized water was added, and the mix was dispersed evenly and poured into a 250 mL beaker. A total of 40 mL of deionized water, 0.016 g of TEMPO catalyst and 0.1 g of NaBr were added and then stirred. Then, 10 mmol of NaClO solution was added slowly and the pH of the solution was more than 10. After stirring for 5 h at room temperature, the pH decreased during the reaction process. We then adjusted the system pH using 0.5 mol/L NaOH solution to keep it stable at 10. After the reaction, the products were washed with deionized water several times to achieve neutrality and to obtain TEMPO oxidized cellulose (TOCN), which was poured into a high-pressure homogenizer at 100 MPa to induce circulating homogenization for 20 min to obtain the nanocellulose dispersion. Subsequently, the concentration of TOCN was adjusted to 0.5 wt% and stored in a refrigerator at 4 °C for further use.

### 2.3. Preparation of PVA Solution

PVA (1.0 g) was dissolved in 100 mL of deionized water and then heated to 95 °C and stirred until completely dissolved. It was then kept at room temperature for further use.

### 2.4. Preparation of Biomimetic GO/TOCN/PVA Aerogel (b-GTPA)

The TOCN suspension (5 mg/mL) and PVA solution (10 mg/mL) were mixed at a volume ratio of 2:1. Then, we prepared the GO suspension at a concentration of 5 mg/mL. Different amounts of GO (0 wt%, 5 wt%, 20 wt%, and 30 wt% of suspension) were added and stirred for 2 h. Then, sulfuric acid solution (1.0% by volume) was gradually added to the suspension of the GO/TOCN/PVA mixture until the pH of the suspension was 4–6. Then, 0.2 mL of the crosslinking agent glutaraldehyde solution (25% by mass fraction) was added to the prepared mixed suspension and stirred continuously for 1 h. Finally, bubbles were removed ultrasonically for 1 h in an ice bath and the suspension was crosslinked in a vacuum oven at 75 °C for 3 h and was then was cooled to room temperature. The homogeneous suspension was then poured into a homemade mold (Figure S1) to obtain the oriented biomimetic structures. The solution was placed on the surface of a cold finger for 20 min to ensure that it was completely frozen. Finally, the forean frozen solution was transferred to a vacuum freeze-drying machine and then freeze-dried at −50 °C for 48 h to obtain biomimetic GO/TOCN/PVA aerogel (b-GTPA). For comparison, the random structure the GO/TOCN/PVA aerogel (r-GTPA) was prepared without a PDMS wedge using traditional unidirectional freezing.

### 2.5. Modification of b-GTPA to Prepare Superhydrophobic b-MGTPA (Modified GO/TOCN/PVA Aerogel)

The GTPA was put into a sealed container and a small vessel containing 8 mL of DDTS was placed at the bottom of the sealed desiccator. Then, it was heated in an oven at 175 °C for 10 h under vacuum to graft the DDTS to the hydroxyl groups of GO, PVA, and TOCN through a silanization reaction to acquire modified GO/TOCN/PVA aerogels (MGTPA).

### 2.6. Characterization

The microstructures of the three different types of aerogels were characterized using scanning electron microscopy (SEM) (ZEISS EVO18, ZEISS, Oberkochen, Germany) images. Fourier transform infrared spectroscopy (FTIR) (Nicolet IS50, Thermo Electron Corp., Madison, WI, USA) was used to record the FTIR data in a range of 400−4000 cm^−1^. Raman spectra were recorded in the range of 100–3500 cm^−1^. The contact angle measurements were performed at room temperature with a Dataphysics OCA 15 optical contact angle measuring system using the sessile drop method (Dataphysics, Stuttgart, Germany).

### 2.7. Density and Porosity

An electronic and digital caliper was used to measure the quality and size of the aerogel, and Equation (1) was used to calculate the density of the aerogel.

(1) ρ=MV

M is the aerogel mass and V is the aerogel volume [24].

According to the solid density of each component and their weight ratios, the density of the solid material is calculated according to Equation (2).

(2) $$\rho_{s}=1/\left(W_{\mathrm{TOCN}}/\rho_{\mathrm{TOCN}}+W_{\mathrm{PVA}}/\rho_{\mathrm{PVA}}+W_{\mathrm{GO}}/\rho_{\mathrm{GO}}+W_{\mathrm{silane}}/\rho_{\mathrm{silane}}\right)$$

where W is the weight percentage of the different components. TOCN, PVA, GO, and silane are the solid densities of TOCN, PVA, GO, and silane, respectively. According to previous literature, the densities of TOCN, PVA, GO, and silane used in this study were 1460, 1269, 2100, and 1273 kg/m^3^, respectively.

The porosity (P) of aerogels was calculated according to Equation (3).

(3) P%=(1−ρρs)×100%

### 2.8. Mechanical Property Measurements

After the aerogel absorbs the oil, the aerogel and oil can be recovered by mechanical extrusion. In order to study the mechanical properties of aerogel, the compressive properties of MGTPA were tested using an Instron 5565 universal material tester (Instron, Boston, MA, USA). The testing method was: placing the aerogel on a universal material testing machine, and then setting the strain value and the relevant test parameters to test at room temperature.

### 2.9. Absorption Capacity and Reusability

The absorption capacity of the aerogels was measured by immersing a piece of the aerogel in an organic liquid until the saturation state was reached. The saturated aerogels were then weighed and the organic solvent absorption capacity (g/g) of the aerogels was calculated as:

$$Q=\frac{m_{\mathrm{sa}}-m_{o}}{m_{o}}$$

where Q is absorption capacity, and m_sa_ and m_o_ are the weights of the aerogel saturated with organic liquids and the pristine aerogel, respectively.

The reusability of b-MGTPA was evaluated via distillation and squeezing of the oil and the removal rate after 10 absorption–desorption cycles was determined. The desorption process was carried out by means of a simple and convenient squeezing method where the mechanism of squeezing was performed for b-MGTPA for 10 cycles.

## 3. Results and Discussion

### 3.1. MGTPA Fabrication and Chemistry Characterization

**Figure 1:**
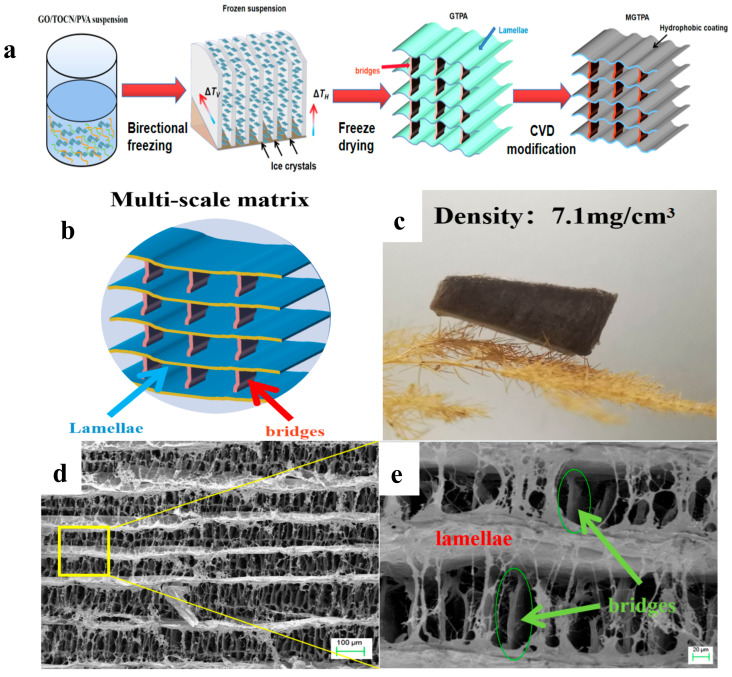
(**a**) Schematic illustration of the fabrication process of b-MGTPA. (**b**) Schematic of the b-MGTPA with a mineral bridge structure. (**c**) Photograph of an ultralight b-MGTPA located on a dogstail grass. (**d**,**e**) SEM images showing the architecture of the biomimetic aerogel at different magnifications.

*Thalia dealbata* stem, with a three-dimensional (3D) architecture of oriented lamellar layers of multi-scale structures that are parallel to the growth direction, which has interconnected bridges, is a lightweight, strong and resilient natural porous material [22]. In order to mimic the architecture of *Thalia dealbata* and the excellent mechanical performance, as shown in Figure S2, cellulose nanofibrils with an average width of 5–20 nm and lengths of up to several micrometers were prepared. We divided the b-MGTPA preparation process into three steps: bidirectional freeze-casting, freeze-drying, and CVD modification. The fabrication scheme of the b-MGTPA is illustrated in Figure 1a. We created a biaxial temperature gradient, including the vertical (ΔT_v_) and the horizontal (ΔT_H_), to obtain the biomimetic structure. During the bidirectional freezing process, the GO/PVA/TOCN suspension was squeezed and repelled by growing ice crystals, and then expelled between the ice layers and the ice crystal gaps, which formed more dendrites that were attached to the lamellar layers (Figure 1b) [25]. Furthermore, with an increase in GO content from 0% to 30%, the density of the MGTPA ranged from 6.48 to 12.83 mg/cm^3^ (Figure S3). Due to the ultralow density, the aerogel was ultralight and could rest on the top dogstail grass without causing any deformations (Figure 1c). Both aerogels exhibited high porosities (>99.21%), low densities, and ultralight characteristics. Scanning electron microscope (SEM) images of the b-MGTPA exhibited a hierarchical mineral bridge structure (Figure 1d,e), successfully mimicking the microstructure of *Thalia dealbata* stem.

**Figure 2:**
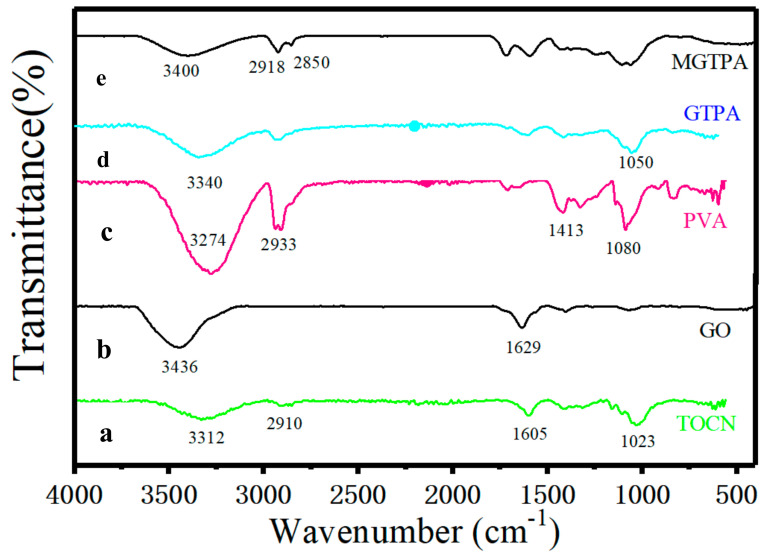
The FTIR spectra of the (**a**) TOCN, (**b**) GO, (**c**) PVA, (**d**) GPTA, and (**e**) MGPTA.

To investigate the chemical structure and CVD modification efficiency of MGTPA, FTIR spectroscopy, Raman spectroscopy tests, and XPS were used. The FTIR spectra of the TOCN, GO, PVA, GPTA, and MGPTA are shown in Figure 2. The spectrum of TOCN (Figure 2a) shows that the absorption peaks at 3312 cm^−1^, 1605 cm^−1^, and 1023 cm^−1^ are attributed to the stretching and bending vibrations of -OH and glycosidic ring vibration, respectively [26], while the vibration at 2910 cm^−1^ corresponds to the stretching vibration of -CH. The spectrum of GO (Figure 2b) occurring at 3436 cm^−1^ and 1629 cm^−1^ corresponds to -OH stretching vibrations and the C=C from an aromatic ring, respectively [27]. The peaks of the PVA (Figure 2c) infrared spectra at 2923 cm^−1^ were associated with the stretching of -CH. The strong absorption peaks at 3280 cm^−1^, 1413 cm^−1^, and 1080 cm^−1^ are characteristic peaks of hydroxyl groups caused by O–H stretching vibration, in-plane bending vibration of CH–OH, and stretching vibration of C–O, respectively. The spectrum of GPTA (Figure 2d) showed similar characteristic peaks, but showed a slightly weakened peak value, indicating the strong interface interaction between the three components of TOCN, PVA, and GO. The weakness of the peak at 3359 cm^−1^ revealed that the compatibility of the GA and PVA chains led to a decrease in the number of free hydroxyl groups, which may indicate the formation of acetal bridges and the completion of acetal reactions [28].

**Figure 3:**
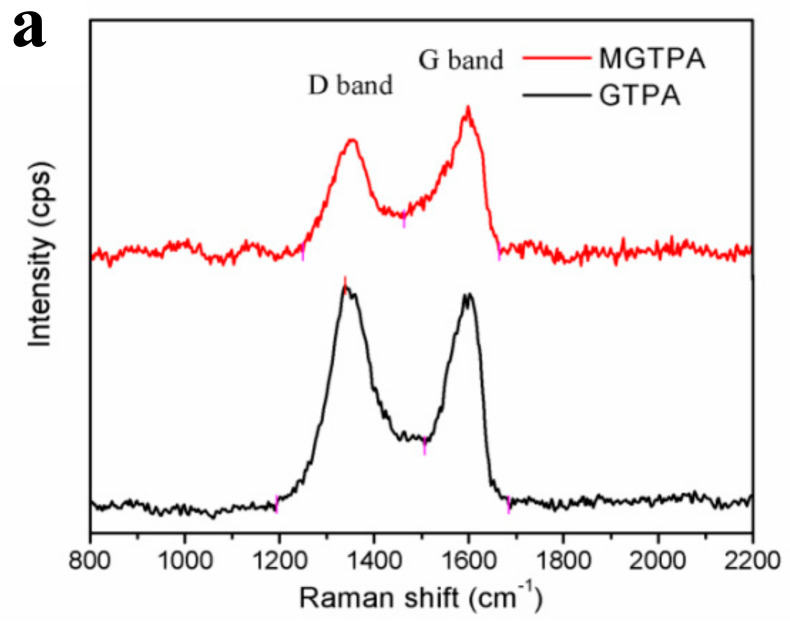

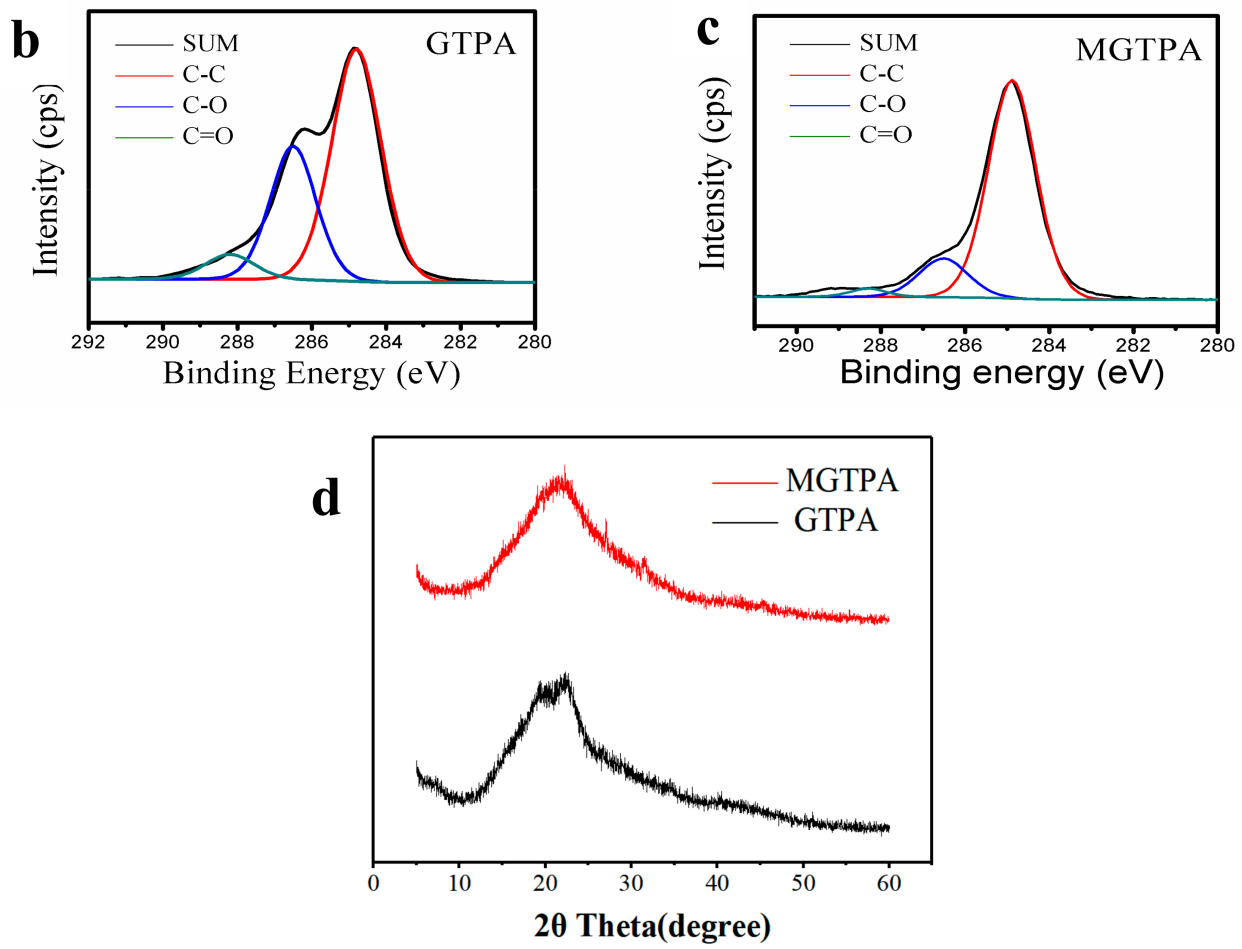
(**a**) Raman spectra of MGTPA and GTPA. XPS high-resolution C1s scans of (**b**) GTPA and (**c**) MGTPA. (**d**) XRD patterns of GTPA and MGTPA.

Figure 3a shows that the I_D_/I_G_ value decreased from 1.41 to 0.87 after CVD modification. The reason for this change may be that the high temperature (175 °C), not only the partially removed the oxygen-containing groups on GO, but also benefitted an increase in graphitization degree, which is favors water–oil separation.

To further confirm that the DDTS was grafted to the hydroxyl groups during CVD modification, X-ray photoelectron spectroscopy was carried out (Figure S4). We detected carbon and oxygen from GTPA, while MGTPA showed silicon peaks from DDTS. The curves were Gauss fitted to indicate the proportion of different bonds (shown in Table 1). The high-resolution C1s core-level spectra show that the proportion of C-C bonds increased from 63.2% to 83.3% due to the grafting of long carbon chains (Figure 3b,c). In the modified TOCN/PVA/GO spectrum (Figure 2e), the intensity of the –CH_2_ bonds (2850 and 2918 cm^−1^) increased, while the intensity of the -OH (3400 cm^−1^) bonds decreased, indicating the grafting of hydrophobic carbon chains after CVD modification, which is in agreement with the XPS results.

**Figure 4:**
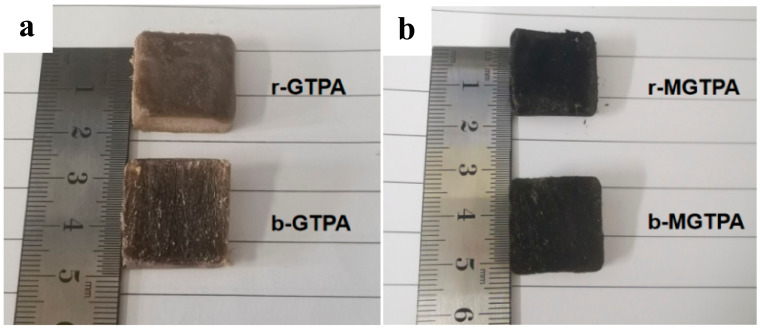
(**a**) Photograph of r-GTPA and b-GTPA. (**b**) Photograph of r-MGTPA and b-MGTPA.

The XRD pattern of the GTPA exhibited a diffraction peak at 19.5° and 22.6° (Figure 3d), corresponding to the diffraction peaks of the PVA and TOCN. Moreover, the peak of GO (10.9°) disappeared, probably due to the relatively small amount of GO that was added to the sample, and the GO was uniformly dispersed in the matrix without agglomeration. After CVD modification, a broad peak was observed at 21.6, which meant that reduced graphene oxide was formed. Simultaneously, the color of the aerogel also changed from brown to black after modification, as shown in Figure 4a,b, which is a good indicator of the reduction of GO. Simultaneously, there was a very low and almost negligible dimensional shrinkage after modification, indicating the good dimensional stability of b-MGTPA.

### 3.2. Mechanical Properties of MCGA

**Figure 5:**
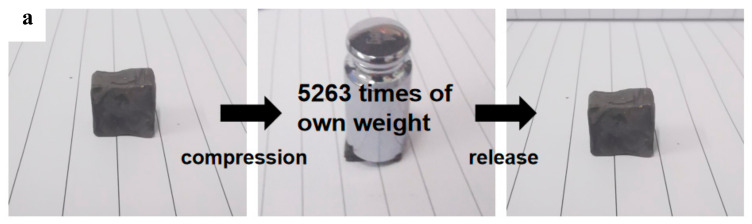

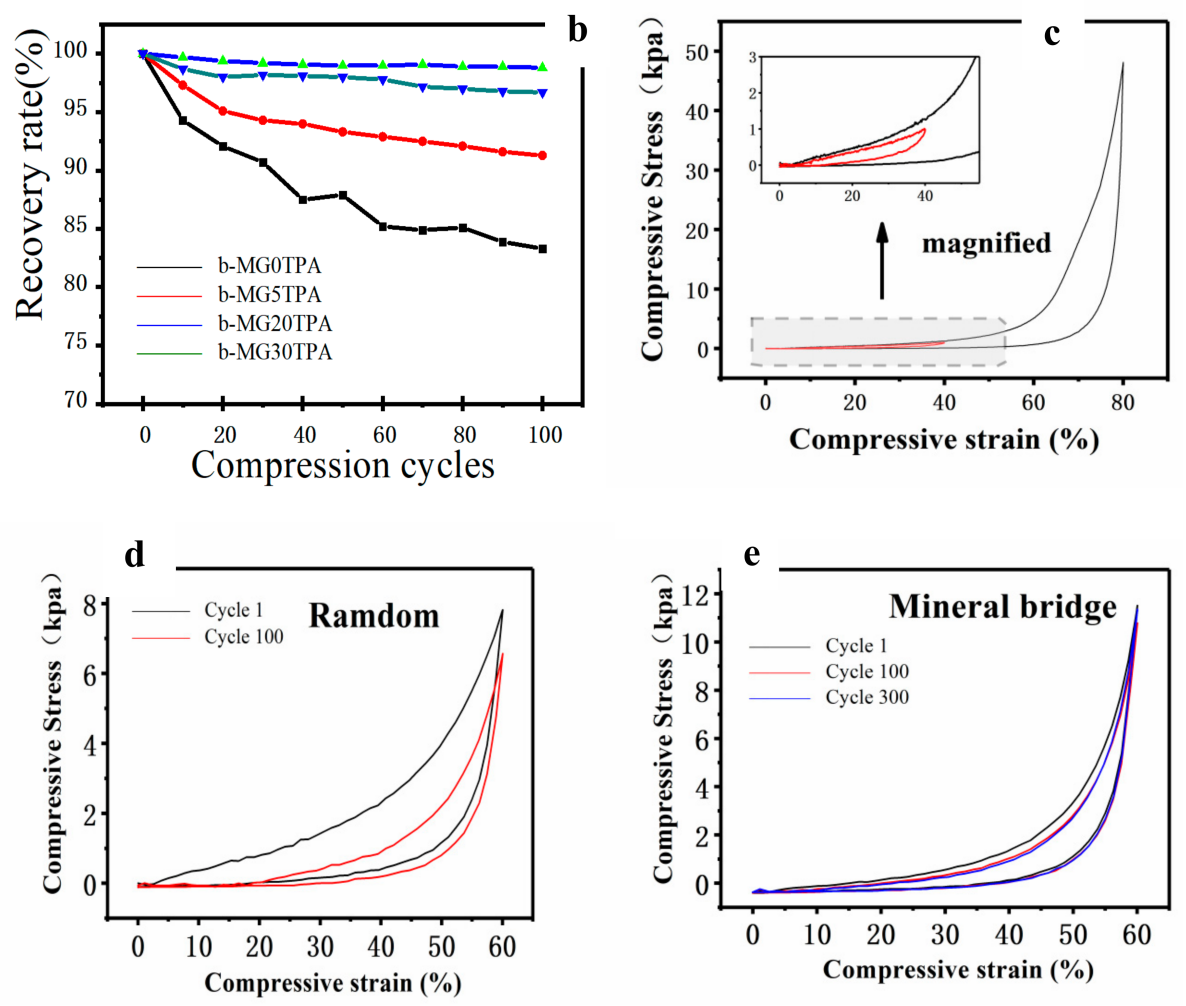
(**a**) Demonstration of the high compressibility and recoverability of MGTPA. (**b**) Recovery rate of different aerogels in cyclic compression tests. All samples were compressed to 60% strain and released to 0% strain for 100 cycles. (**c**) Stress−strain curves of b-MG_20_TPA at 40% and 80% strain. Comparison between (**d**) random and (**e**) biomimetic structure aerogels in their compression−recovery behaviors.

Mechanical properties, especially reversible compressibility, are particularly important for the evaluation of adsorbed materials to obtain a high oil-recovery rate from squeezing. To evaluate the mechanical properties of MGTPA, a series of compression tests were performed. Figure 5a shows a typical photograph of b-MGTPA, which can withstand pressures more than 5000 times its own weight (where it is compressed to 5% of the original height). After the load is removed, the b-MGTPA can quickly return to its initial height without any damage, and the structure exhibits a high compression resilience and suffers no obvious lateral deformation in the process of compression.

In addition, through uniaxial compression and cyclic loading tests, we studied the stress–strain curve of MGTPA as well as its fatigue resistance stability. As shown in Figure 5b, a universal tension machine was used to load the MGTPA at a speed of 100 mm/min, and the MGTPA was compressed to 60% for 100 and 300 cycles. We investigated the effect of GO content on the mechanical properties. Comparing MGTPA with different GO concentrations (0 wt%, 5 wt%, 20 wt%, and 30 wt% of suspension), Figure 4b shows that the recovery rate obviously improved after adding a certain amount of GO, which might be attributable to the rigidity of the GO sheets in the TOCN/PVA matrix. In addition, MG_20_TPA showed the highest original strength of 98.4% after being compressed for 100 cycles, and the highest stress-retention rate (it retained 99.3% and 98.4 of its maximum stress) after 100 and 300 cycles (Figure S5), which exhibited an almost negligible plastic deformation. Therefore, we selected MG_20_TPA for the following mechanical property test.

Figure 5c shows the cyclic compressive loading process of the b-MG_20_TPA with different strains (40% and 80%). There were two obviously different regions during the compression–release process. An elastic deformation region appeared under a low compressive strain (less than 45%), followed by a densification region under a high compressive strain (more than 45%). The stress increased linearly with strain due to the elastic bending of the bridge when the strain was below 45%. At a strain of more than 45%, the stress increased rapidly with the strain, producing a larger compressive stress and hysteresis loop due to the collisions between the lamellar frames.

To investigate the excellent mechanical strength of the biomimetic MG_20_PTA, we systematically compared it with MG_20_PTA with random and biomimetic architectures, as shown in Figure 5d. The b-MG_20_PTA showed a maximum compressive stress of 11.51 kPa when it underwent 60% compression. The 100 and 300 cycle stress–strain curves compared with the first curve almost remained the same. r-MG_20_TPA was much less robust and resilient. With a maximum compressive stress of 7.82 Kpa, after only 100 compressive cycles at 60% strain, the maximum stress of the aerogel already decreased obviously, retaining only 83.1% of its maximum stress.

**Figure 6:**
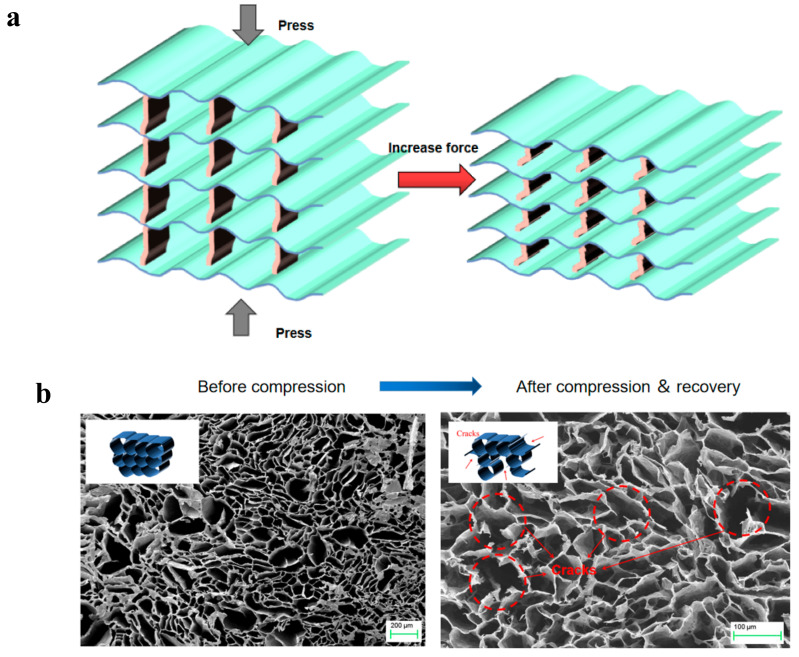
(**a**) Schematic diagram of the mechanism of the high compressibility and recoverability of biomimetic mineral bridge structures. (**b**) SEM image showing the r-MGTPA with random structure before and after 100 cycles of compression (strain = 60%) and recovery. Inserts show the corresponding schematics.

In the compression process, stress can be evenly distributed across the lamellar layer through the bridge to avoid stress concentration, resulting in permanent deformation and structural damage to the material (Figure 6a). The bridge between the lamellar layers acts as an infinite number of small “springs”, prompting elastic deformation during the compression cycle. When the external force is removed, biomimetic aerogels are able to fully recover their original shape without significant deformation or damage. Compared to bionic structures, random structures have more concentrated stress areas, so the compression performance ultimately depends on the weakest connection. In the compression process, the weakest part is more likely to rupture, resulting in inelastic deformation (Figure 6b), which makes causes the recovery performance to be poor, when the external force is removed, and it cannot fully recover.

The above tests indicated that b-MGTPA was much more resilient than r-MGTPA due to the unique architectures of the “lamellar layers with interconnected bridges”. The above results explain that b-MGTPA possesses excellent mechanical properties due to its unique “lamellar layers with interconnected bridges” and there is hope for it to be applied in sensors, electrode materials, and flexible electronics in the future.

### 3.3. Absorption Properties and Reusability

**Figure 7:**
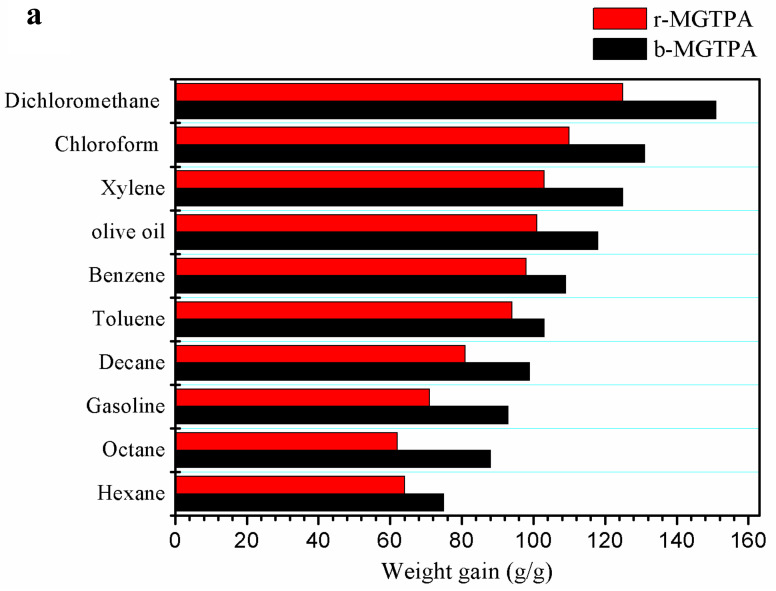

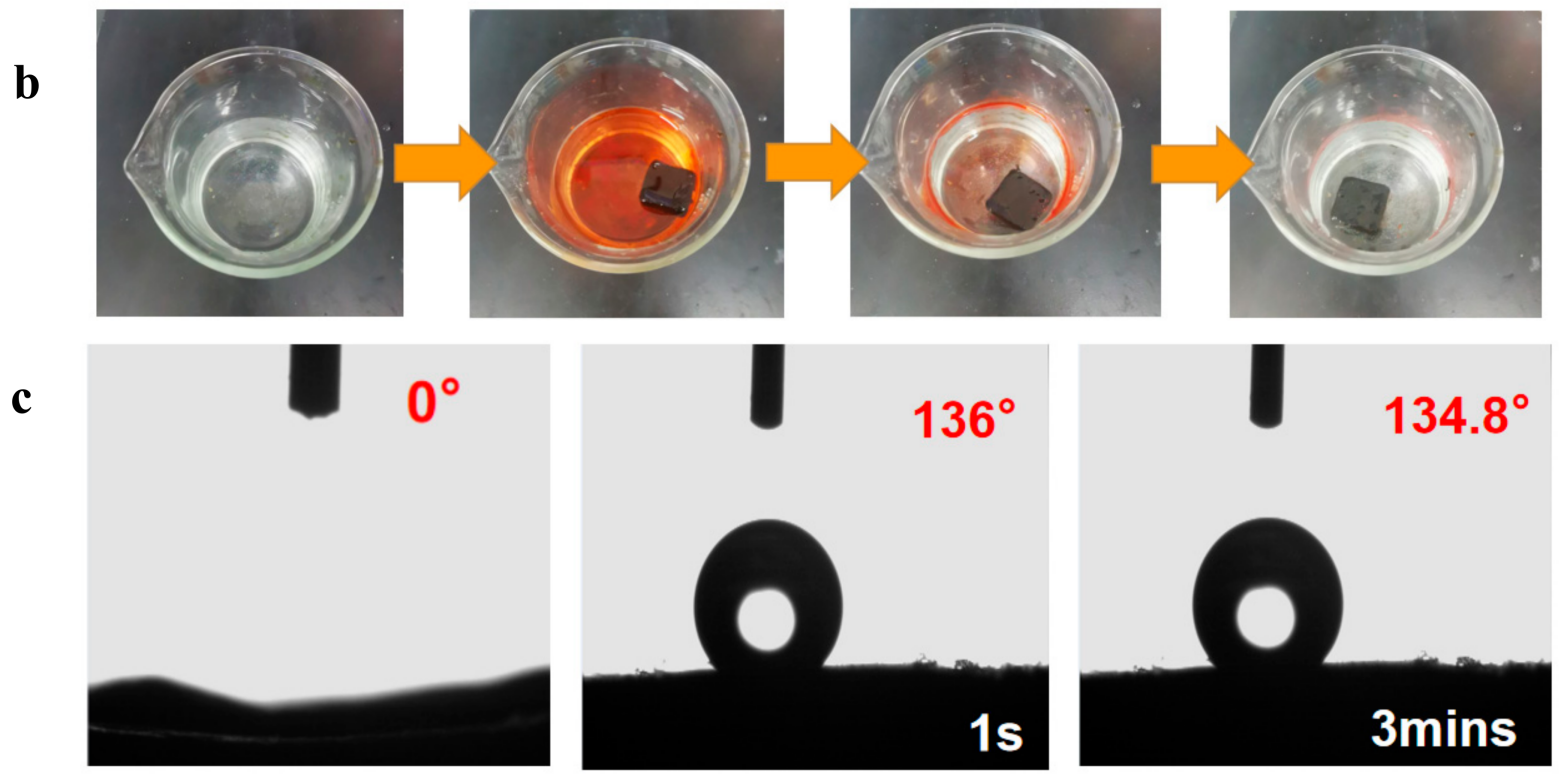
(**a**) Absorption capacity of d-MG_20_TPA and b-MG_20_TPA for various solvents. (**b**) Demonstration of selective oil (dyed red with Sudan III) absorption atop water using b-MGTPA. (**c**) Water contact angle of the b-GTPA and b-MGTPA taken at 1 s and 3 min.

Due to it low density, high compressive, and high porosity, MGTPA has great application prospects for absorbing oil and organic pollutants. In order to systematically study the absorption capacity of MGTPA, MGTPA was used for the saturated absorption of various oils and organic solvents, such as hexane, olive oil, toluene, and some common pollutants and leaks in daily life or in the chemical industry. As shown in Figure 7a, the saturated absorption capacity of b-MG_20_TPA varies from 75 to 151 times its own weight for various oils, depending on the density, viscosity, and surface tension of the absorbed oil. In addition, biomimetic structures have a better oil adsorption performance than random structures, which may be due to the fact that the unique mineral bridge structure can provide greater space for oil absorption.

As shown in Figure 7b, MG_20_TPA could be used to absorb and separate hexane floating on the surface of water and it can be seen that the hexane dyed using Sudan red III was rapidly and completely absorbed by MG_20_TPA, accomplishing the separation of hexane and water. Due to the ultralight and hydrophobic coating of the aerogel, it still can float on the water’s surface, even if saturated, which is of high value in practical applications and facilitates unified collection after adsorption.

In order to further determine the contact angle of MGTPA, we use a contact angle tester for modified GTPA. As can be seen from Figure 7c, the contact angle was 0° before modification and was not hydrophobic. The contact angles of the modified aerogel to water at 1 s and 180 s were 136° and 134.8°, respectively, greater than 90°, which exhibited surface hydrophobicity.

**Figure 8:**
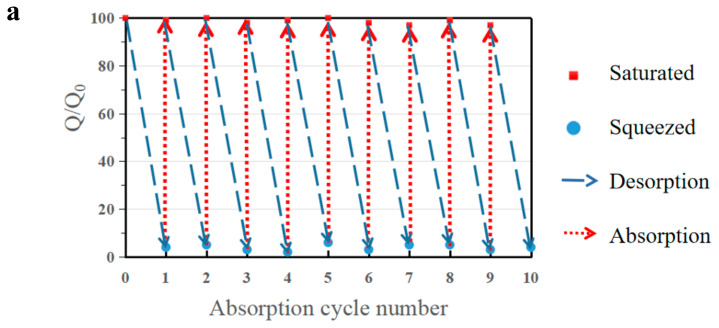

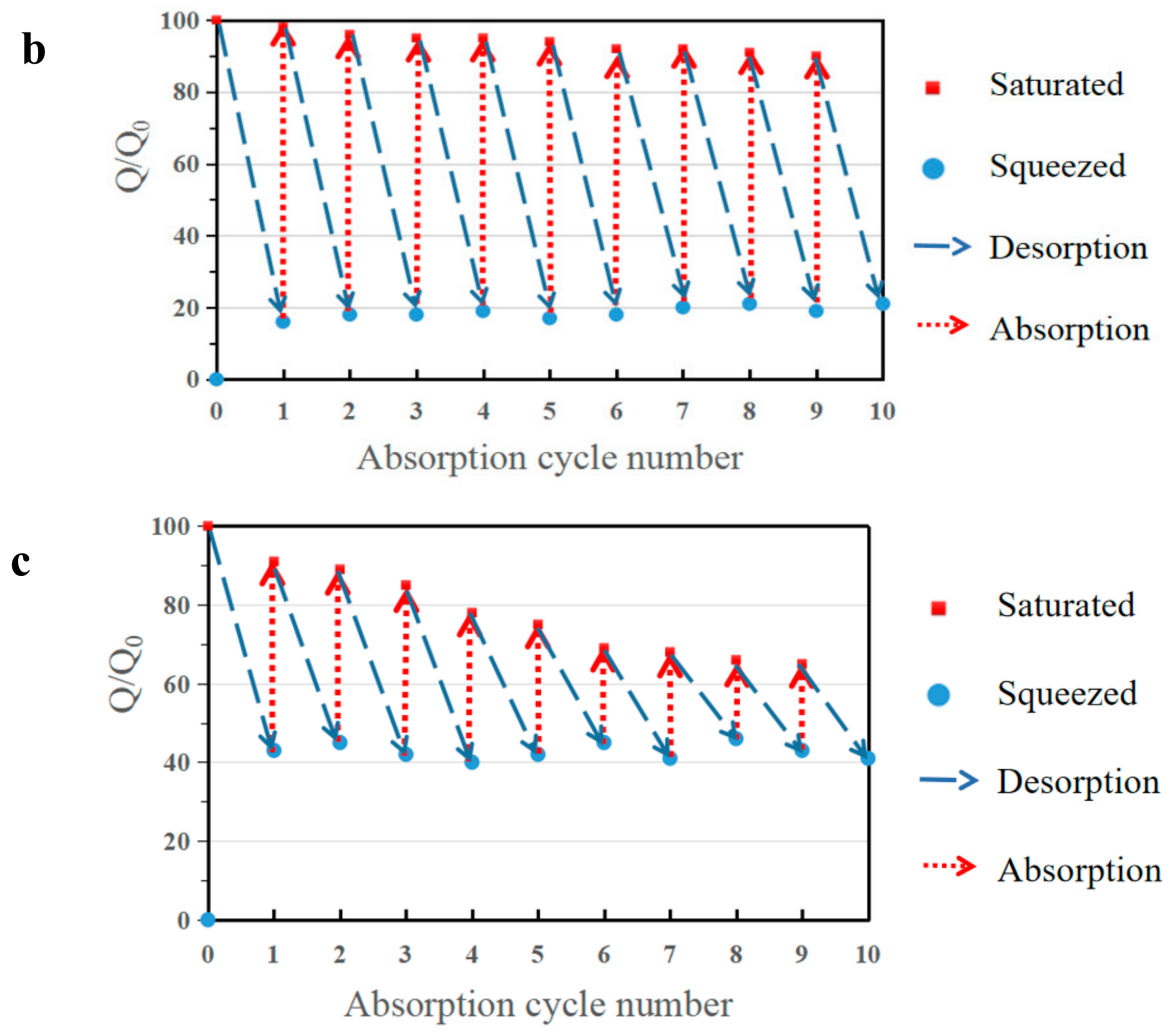
(**a**) Oil recovery ability of b-MG_20_TPA over 10 cycles of absorption and desorption by distillation of hexane. Ethanol was selected as the model oil to test the distillation. Demonstration of expelling absorbed hexane by squeezing from (**b**) b-MG_20_TPA and (**c**) r-MG_20_TPA. Oil recovery ability of b-MG_20_TPA and r-MG_20_TPA over 10 cycles of absorption and desorption by mechanical squeezing.

As a comprehensive and suitable oil–water separation and absorption material, Absorbent can not only effectively separate oil and water from an oil–water mixture, but it can also efficiently recycle the absorbed organic solvent. The reusability and oil recovery of adsorbent materials are a significant standard for evaluating the application of adsorbent materials. In this work, b-MGTPA proved to have excellent hydrophobic properties and compressibility, which grants it two methods to recycle absorbed oil or organic solvent in actual oil–water separations according to different practical needs. First, when dealing with low boiling point oils, such as hexane or toluene, the absorption/distillation cycle is an option. Saturated b-MGTPA is heated to near the boiling point of the solvent to release the absorbed oil, and the oil can be recovered by collecting the evaporating oil vapor. This kind of recycling efficiency is very high, and can basically achieve a 98% recycling rate after 10 cycles of absorption and desorption (Figure 8a).

When we need to address expensive oils or retrieve high-boiling point oils, we can use the mechanical characteristics of b-MGTPA to conveniently recycle oil through simple mechanical squeezing. The cyclic absorption–extrusion behavior of MGTPA was studied using hexane as the model adsorbent. As a result, the biomimetic structure could maintain more than 92% of its initial saturated absorption capacity to a large extent after the 10th cycle test (Figure 8b), which indicated that the porous structure of b-MGTPA was not destroyed and maintained property stability in the absorption and squeezing process. In contrast, the random structure underwent huge losses (only maintained 60–75% of its initial capacity) after 10 cycles of absorption and squeezing (Figure 8c), which was consistent with the results of scanning electron microscopy. Furthermore, the b-MGTPA showed around 15–20% residual oil rate in the cycle, which was higher than other reported compressible absorbents, which usually retain about 40–60% oil after squeezing. The above results clearly demonstrate the excellent reusability of b-MGTPA, which has a high compressibility and high oil recovery ability though mechanical squeezing that has not been achieved by other aerogels, as shown in Table 2, indicating that distillation and squeezing can be used to deal with various situations of oil–water separation from accidents. b-MGTPA can be selected as an ideal high-performance oil sorbent candidate for practical use.

## 4. Conclusions

A biomimetic highly compressive and hydrophobic graphene/polyvinyl alcohol/cellulose nanofiber aerogel with unique three-dimensional (3D) interconnected lamellar layers with bridges was successfully prepared through bidirectional freezing. Long carbon chains were grafted to the surface of GTPA, which yielded the modified GTPA (MGTPA) hydrophobicity (WCA = 134°) through CVD modification.

b-MGTPA exhibits an excellent recovery rate of 98.4% and a maximum stress of 99.3% from 60% strain after 100 cycles, due to its unique structure of lamellar layers with bridges, which act as an infinite number of small “springs”. In addition, b-MGTPA also shows a high absorption capacity, 75–151 times its weight, which was tested for various oils and chemical solvents, and that surpasses most hydrophobic cellulose-based aerogels. The adsorption capacity of b-MGTPA decreases less than 10% during squeezing and less than 2% during distillation after 10 cycles, indicating a high degree of reusability. Approximately 80–85% of absorbed oil can be recovered by simple mechanical extrusion. b-MGTPA has great application potential in cleaning water spills and as organic solvents due to its characteristics of being hydrophobic, and having a low cost, simple preparation and, stable performance.

## Figures and Tables

**Table 1:** The proportion of different bonds of XPS.

	GTPA		MGTPA	
	Position	%	Position	%
C–C	284.7	63.2	284.8	83.3
C–O	286.4	30.1	286.5	14.3
C=O	288.3	6.7	288.2	2.4

**Table 2:** Performance comparison of b-MGTPA with 3D absorbent materials reported in the literature.

	Absorbent Material	Density (mg/cm^3^)	Absorption Capacity (g/g)	Compressibility (%), Oil Recovery (%)	Reference
1	CNF/PVA/MTMS aerogel	10.2	45–99	Compressible to 70% 55% oil recovery	[11]
2	freeze-dried SA/GO aerogel	20	20–50	Compressible to 80% 80% oil recovery	[29]
3	Carbon fiber aerogel from bamboo	4.3–8.3	22–80	Compressible and 60% 50% oil recovery	[30]
4	CNF/PVA/MMT aerogel	26.25	40–68	Not compressible	[31]
5	lignin, agarose, and PVA	0.052	44–96	Compressible and 60% 60% oil recovery	[32]
6	Directionally freeze-dried TOCN/GO/PVA carbon aerogel	6.17	155–288	Compressible to 50% and 50% oil recovery	[9]
7	Directionally freeze-dried	4.3–8.3	120–200	Compressible to 50% and 50% oil recovery	[6]
8	GO/TOCN aerogels	6.8	100–240	Compressible and 50% oil recovery	[33]
9	Freeze-dried CNF/graphene	7.5	44–265	Compressible to 50% and 80% oil recovery	[5]
10	Directionally freeze-dried TOCN/GO/PVA aerogels	7.1	75–151	Compressible to 90% and 85% oil recovery	This work

## Data Availability

Data sharing not applicable.

## Supplementary Materials

The following supporting information can be downloaded at: https://www.mdpi.com/article/10.3390/polym14061077/s1, Figure S1: Schematic illustrations of bidirectional freezing; Figure S2: TEM image of prepared TOCN; Figure S3: The density of the b-MGTPA as a function of initial GO concentration; Figure S4: XPS survey scans of GTPA and MGTPA; Figure S5: Comparison between MGTPA with different GO concentrations in their compression−recovery behaviors.
