# Supplementary material for: Biomimetic, Highly Reusable and Hydrophobic Graphene/Polyvinyl Alcohol/Cellulose Nanofiber Aerogels as Oil-Removing Absorbents
Source: Polymers (Basel). 2022 Mar 8;14(6):1077. doi: 10.3390/polym14061077 (PMC8951047; doi:10.3390/polym14061077)
Supplement: Supplementary file 1 [file polymers-14-01077-s001.zip › polymers-1508080-supplementary.pdf]

First,the GO/PVA/TOCN suspension was poured into the homemade molds.In order to generate a bidirectional temperature gradient, a PDMS-wedge with low thermal conductivity at a specific Angle(15°) is added between the suspension and the cooling copper block.Except for the large temperature gradient in the vertical direction（ΔT_H）.There is also a temperature gradient at both ends of the wedge（ΔT_v）,due to the existence of PDMS wedge.The ice crystals grew directionally with gradient along both horizontal direction and vertical direction，which formed paralleled and ordered ice columns.

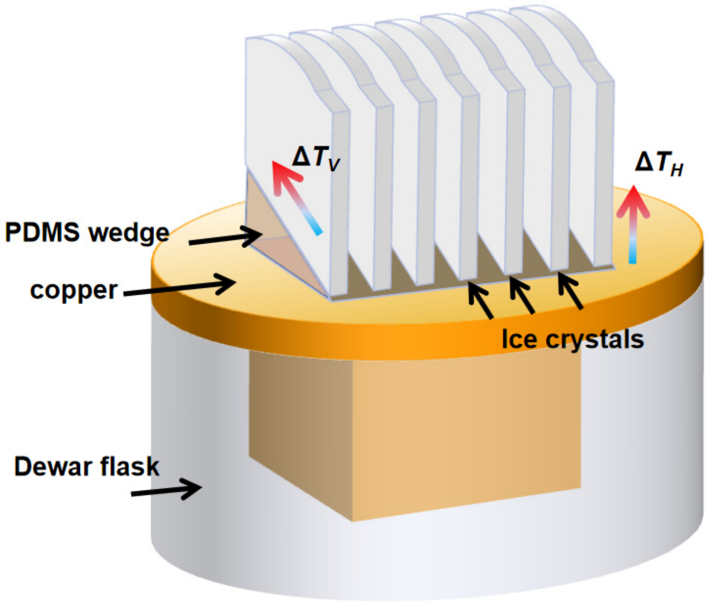

Figure S1. Schematic illustrations of bidirectional freezing.

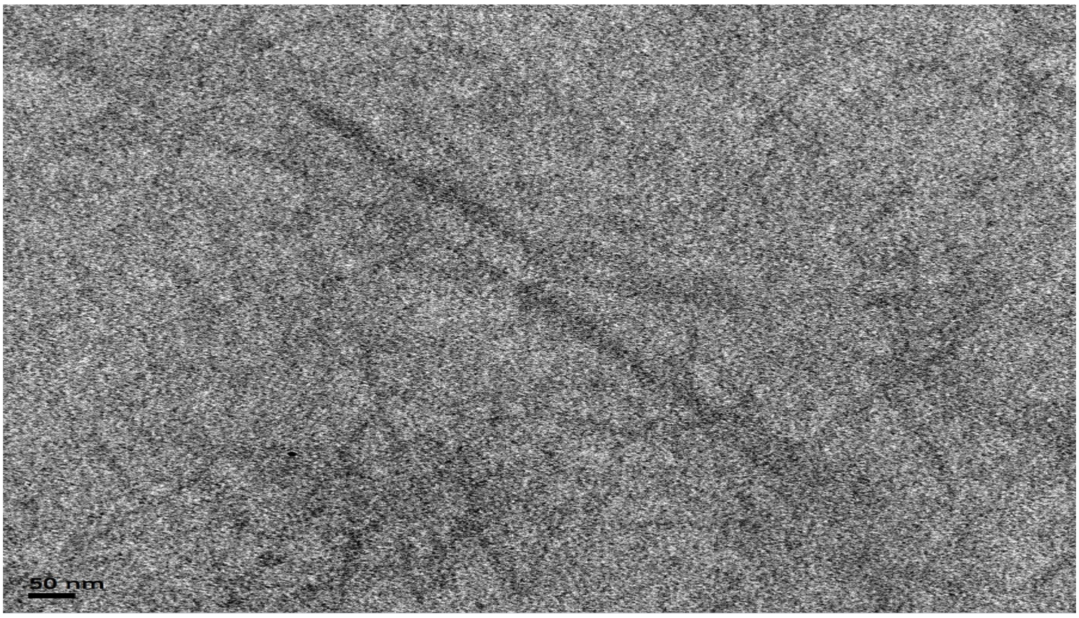

Figure S2. TEM image of prepared TOCN.

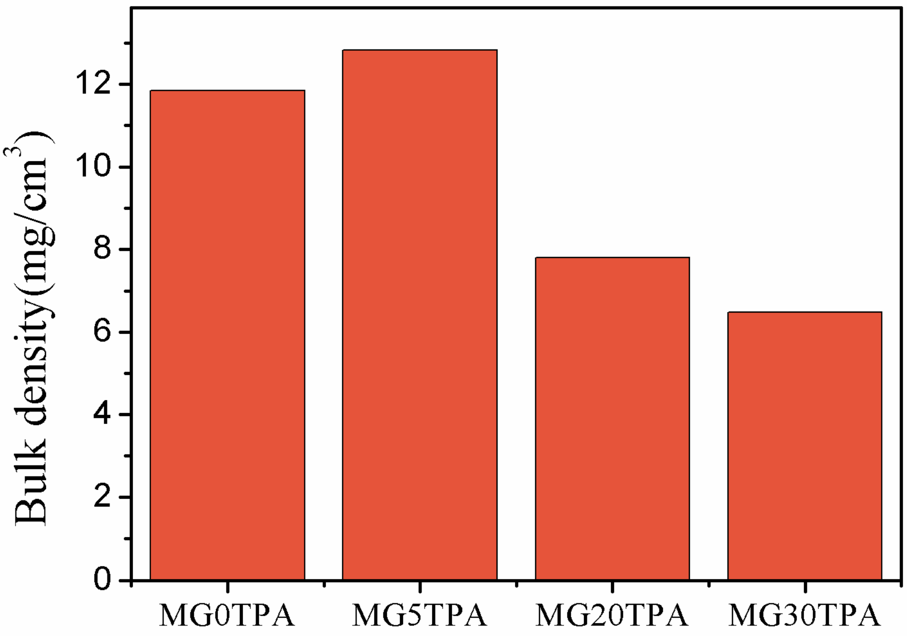

Figure S3. The density of the b-MGTPA as a function of initial GO concentration.

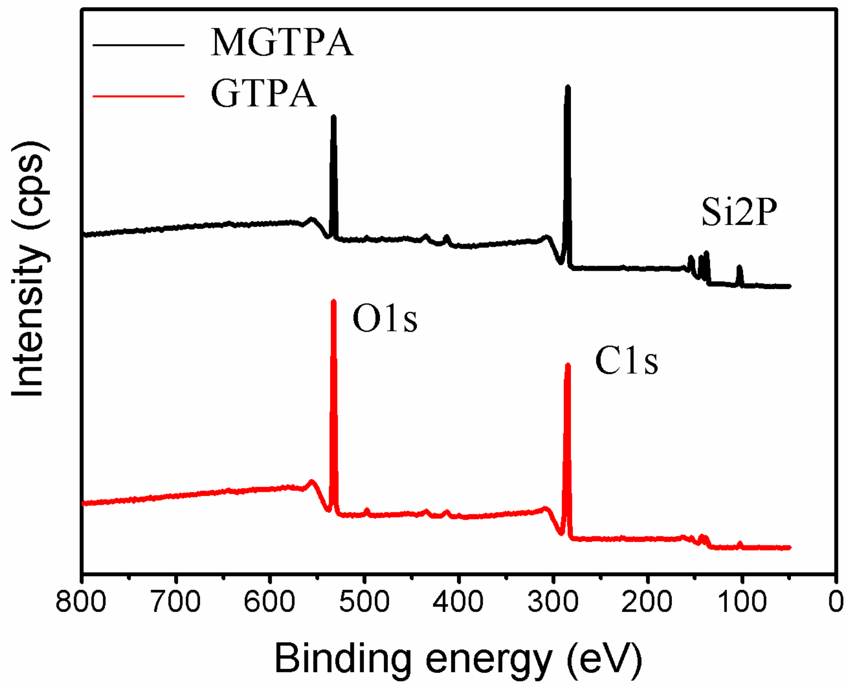

Figure S4. XPS survey scans of (a) GTPA and (b) MGTPA. The peak intensity of the C1s peak greatly increased after CVD modification with n-dodecyltriethoxysilane (DDTS), thus indicating grafting of the long carbon chains. Silicon peaks were also detected in MGTPA via XPS.

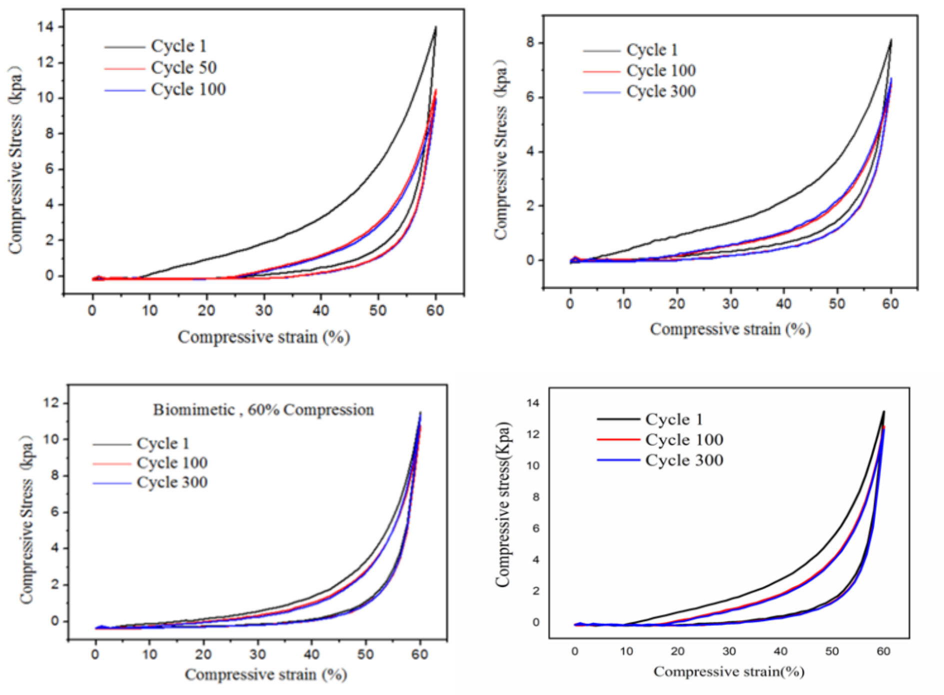

Figure S5. Comparison between MGTPA with different GO concentrations in their compression−recovery behaviors (a) MG$_0$TPA,(b) MG$_5$TPA,(c) MG$_{20}$TPA,(d) MG$_{30}$TPA.When compressed to 60%, the maximum stresses of the MG$_0$TPA,MG$_5$TPA,MG$_{20}$TPA and MG$_{30}$TPA are 14.06,8.15,11.51 and 13.5 kPa, respectively. (c) The aerogel retains 71.6% ,82.5%,99.3% and 97.4% of its original strength after 100 cycles, respectively.
